# Correction: Genetic susceptibility to angiotensin-converting enzyme-inhibitor induced angioedema: A systematic review and evaluation of methodological approaches

**DOI:** 10.1371/journal.pone.0227056

**Published:** 2019-12-19

**Authors:** Haivin Aziz Ali, Anne Fog Lomholt, Seyed Hamidreza Mahmoudpour, Thorbjørn Hermanrud, Anette Bygum, Christian von Buchwald, Marianne Antonius Jakobsen, Eva Rye Rasmussen

The third author’s initials appear incorrectly in the citation. The correct citation is: Ali HA, Lomholt AF, Mahmoudpour SH, Hermanrud T, Bygum A, von Buchwald C, et al. (2019) Genetic susceptibility to angiotensin-converting enzyme-inhibitor induced angioedema: A systematic review and evaluation of methodological approaches. PLoS ONE 14(11): e0224858. https://doi.org/10.1371/journal.pone.0224858.
