# Supplementary material for: Genetic susceptibility to angiotensin-converting enzyme-inhibitor induced angioedema: A systematic review and evaluation of methodological approaches
Source: PLoS One. 2019 Nov 11;14(11):e0224858. doi: 10.1371/journal.pone.0224858 (PMC6844479; doi:10.1371/journal.pone.0224858)
Supplement: S1 Table — (DOCX) [file pone.0224858.s001.docx]

**S1 Table**. Details on individual scores for the included studies based on Q-genie tool

| **Study** | **Question** | | | | | | | | | | | |  | |
| --- | --- | --- | --- | --- | --- | --- | --- | --- | --- | --- | --- | --- | --- | --- |
|  | **Rationale for study** | **Selection and outcome** | **Comparability of comparison group** | **Technical exposure** | **Non-technical exposure** | **Other sources of bias** | **Sample size and power** | **A priori planning of analysis** | **Statistical methods and control of confounding** | **Testing of assumptions and inferences for genetic analysis** | **Appropriateness of inferences drawn from results** | **Score** | |  |
| **Bas 2009 [16]** | 5 | 5 | 4 | 4 | 3 | 4 | 2 | 4 | 3 | 4 | 4 | 42 | |  |
| **Duan 2005 [22]** | 5 | 3 | 4 | 4 | 2 | 3 | 3 | 4 | 3 | 4 | 4 | 39 | |  |
| **Gulec 2008 [17]** | 6 | 5 | 3 | 2 | 4 | 3 | 2 | 3 | 2 | 3 | 3 | 36 | |  |
| **La Corte 2011 [20]** | 5 | 3 | 3 | 3 | 2 | 3 | 3 | 4 | 3 | 4 | 4 | 37 | |  |
| **Moholisa 2013 [21]** | 6 | 4 | 4 | 4 | 2 | 3 | 2 | 4 | 3 | 4 | 4 | 40 | |  |
| **Pare 2013 [19]** | 6 | 5 | 5 | 6 | 3 | 5 | 4 | 5 | 5 | 6 | 5 | 55 | |  |
| **Woodard-Grice 2010 [18]** | 6 | 4 | 4 | 3 | 3 | 4 | 4 | 4 | 3 | 5 | 5 | 45 | |  |

Q-genie tool: Quality of genetic association studies, the maximum score for each question is seven.
